# Interventions for unpaid carers of people living with breathlessness due to chronic respiratory diseases: Scoping review

**DOI:** 10.1017/S147895152510148X

**Published:** 2026-02-13

**Authors:** Eleanor Rochester, Nikel-Shaniece Hector-Jack, Glenn Robert, Charles Reilly, Matthew Maddocks, Lisa Brighton, Wei Liu, Assma Ibrahimi, Irene Higginson

**Affiliations:** 1 Cicely Saunders Institute of Palliative Care, Policy and Rehabilitation, Florence Nightingale Faculty of Nursing, Midwifery & Palliative Care, King’s College London, London, UK; 2 Methodologies Division, Florence Nightingale Faculty of Nursing, Midwifery & Palliative Care, King’s College London, London, UK; 3 Department of Physiotherapy, King’s College Hospital, London, UK; 4 Department of Engineering, Faculty of Natural, Mathematical and Engineering Sciences, King’s College London, London, UK; 5 Faculty of Life Sciences and Medicine, King’s College London, London, UK

**Keywords:** Unpaid caregivers, breathlessness, COPD, respiratory disease, Interstitial Lung Disease

## Abstract

**Objectives:**

Carers of people living with breathlessness face common challenges due to the chronic, distressing and unpredictable nature of the symptom. These include unmet information and support needs resulting in worsened health and psychosocial outcomes. This review aimed to (1) identify the relative volume of studies on supportive interventions for carers of people living with breathlessness due to different respiratory diseases, (2) characterize the nature of the interventions, and (3) explore their reported effectiveness on outcomes identified by carers as being important.

**Methods:**

Medline and CINAHL were searched for studies reporting interventions targeting unpaid adult carers of people with breathlessness, lung cancer, chronic obstructive pulmonary disease (COPD), Interstitial Lung Diseases (ILD) published 2000-2025. Intervention characteristics and reported outcomes were extracted and compared across diagnoses and intervention categories. Our findings were shaped by consultation with unpaid carers in a series of patient and public involvement workshops.

**Results:**

From 72 included interventions, three approaches were identified: Education, therapeutic support, and interventions for patient management. Interventions for lung cancer carers most frequently offered therapeutic support to the carer, while those for COPD carers most frequently focused on managing the patient. COPD and ILD carers have been underserved by research. We found few therapeutic support interventions for COPD carers. Reporting of carer demographics was poor, including among RCTs.

**Significance of results:**

There was a dominance of research focusing on carers of people with lung cancer (56% of participants). In PPI consultations, carers identified stigma and poor communication with health providers as factors contributing to the disparity between lung cancer and other respiratory diseases. More research is needed to compare the efficacy of different intervention strategies to improve outcomes that matter most to carers. To improve equity, researchers must consistently report carer demographics and prioritize developing interventions for carers underserved by research.

## Introduction

### Caring for someone with breathlessness

Breathlessness is the most common and burdensome symptom in advanced respiratory disease that can worsen over time despite optimal pharmacological treatment of the underlying condition (Global Initiative for Chronic Obstructive Lung Disease 2022). It affects up to 90% of the over 300 million people globally with chronic obstructive pulmonary disease (COPD), interstitial lung diseases, and/or lung cancer. In coming decades, the burden of breathlessness on health and social care systems globally will rise (Adeloye et al. 2022; Luo et al. 2023). Breathlessness results in high formal and informal care costs (Dzingina et al. 2017) and is associated with frequent emergency department presentations and reattendances (Bone et al. 2016; Hutchinson et al. 2017). In recent years leaders in respiratory medicine have highlighted the importance of recognizing chronic breathlessness as a distinct syndrome to focus research and clinical care (Johnson et al. 2017).

Breathlessness is highly distressing not only for the individual experiencing it, but also their family and carers (Gysels and Higginson 2011; Currow et al. 2017). In this review, we use the definition of a carer from Carers UK as “a person who provides unpaid care and support to a family member, friend or neighbor who is disabled, has an illness or long-term condition, or who needs extra help as they grow older” (Carers 2024). A recent mixed methods systematic review found that many carers of people with breathlessness live in a state of heightened anxiety, with the unpredictability of breathlessness leading to high burden, sleep disturbance and psychological distress (Blütgen et al. 2025). Carers experience negative economic and social impacts of their caring role such as a reduction in working hours or loss of social activities (Meier et al. 2011; Malik et al. 2013; Mosher et al. 2013; Badr et al. 2017). In qualitative studies, carers of people living with breathlessness due to advanced disease described the symptom of breathlessness as very psychologically distressing for them, and one they are ill-prepared to manage (Gysels et al. 2007; Gysels and Higginson 2009; Nakken et al. 2015).

### Purpose of this study

A recent systematic review reported high levels of unmet need among carers of people with breathlessness and highlighted the need for more professional support, information on helping the person they are caring for better manage their symptoms, resources to manage their own anxiety, and social needs including for respite from their caring role (Blütgen et al. 2025). Studies of unpaid carers of people living with COPD, lung cancer, and Interstitial Lung Diseases (ILD) have also identified unmet needs relating to symptom management, access and coordination between health professionals, support with daily living, and psychological or emotional support (Caress et al. 2009; Malik et al. 2013; Chen et al. 2016; Lee et al. 2020). Previous reviews have identified some potentially effective strategies for supporting these carers. Blütgen et al. identified 10 studies of interventions targeted to carers of people with chronic breathlessness. The authors summarized benefits to carers from these interventions including improved coping, symptom management, communication skills, and self-care (Blütgen et al. 2025). This previous review primarily described the burden, unmet needs, and coping experiences of carers supporting people with breathlessness. Building on this, the present review focuses specifically on evaluated supportive interventions, systematically mapping the types, content, and reported effectiveness of interventions designed to address carers’ needs to identify evidence gaps and opportunities for more equitable carer support. Before new interventions to meet the unmet needs of these carers are developed, or existing interventions are applied to new populations, it is critical to understand which approaches have been tried previously, among which populations, and what evidence is available on the effectiveness of existing approaches. This review aimed to (1) identify the relative volume of studies on supportive interventions for carers of people living with breathlessness specifically due to chronic respiratory disease, (2) characterize the nature of the interventions with respect to underlying theory, delivery, and reported outcomes and, (3) where data were available explore the reported effectiveness of carer-targeted interventions stratified by underlying diagnosis. The review questions were as follows:

- What evidence exists on interventions for adult carers and family members of people living with breathlessness due to respiratory disease?
- Which carers were targeted for support?
- What interventions have been provided, who delivered them, where were they delivered, and what were the intended outcomes?

## Method

### Guiding framework

Our review followed Arksey and O’Malley’s 6-step scoping review process (Arksey and O’Malley 2005), refined by Levac et al. (2010). The steps are (1) Identify the research question, (2) Identify relevant studies, (3) Select studies, (4) Chart the data, (5) Summarize and report results, and (6) Incorporate consultation with stakeholders. In this study the consultation stage was conducted to explore what mechanisms may be driving differences in research for different groups of carers, and the relevance of review findings to the lives of carers in the UK. This review followed a pre-specified protocol which is available in the supplemental material and is reported in accordance with the PRISMA-ScR checklist (Tricco et al. 2018).

### Identifying relevant studies

PubMed and CINAHL were searched up to April 2025. A full search strategy can be found in supplemental material. Other methods for identification of studies were searches of the Clinicaltrials.gov trial protocol registry, citation screening of NICE evidence reviews relating to adult carers, and citation screening of relevant systematic and scoping reviews found during database searches. These searches were conducted in April and May 2024.

Search results were imported into Covidence and automatically screened for duplicates. Additional duplicates were removed manually during screening.

### Study selection

Carers of asthma patients were excluded because asthma is a reversible airway disease when managed with inhalers and is not life-limiting.

Records were screened for inclusion based on pre-specified criteria (Table 1). All records were screened by 2 reviewers (ER, NH, or AI) at both the title/abstract and full-text stages. Conflicts were resolved by ER, and regular meetings were held to ensure consensus.

**Table 1.** Inclusion criteria

Population	Intervention	Outcome	Other
Adult carers or family members of adults with breathlessness or diagnosis of COPD, Lung cancer, Interstitial lung disease, Mesothelioma, Fibrotic lung disease following SARS-CoV2 infection	Any intervention that includes unpaid carers by design (not patient-focused interventions where carers were incidentally involved) Any comparator permitted	Reported outcomes among unpaid carers or family members	Primary research, published in English, after 2000

### Charting and interpreting the data

A data extraction form was developed in Microsoft Excel and refined throughout the process in line with the iterative nature of scoping reviews (Levac et al. 2010). Charting, consultation, and analysis were conducted in parallel to ensure a dynamic approach to data synthesis.

We combined data from articles that reported on the same intervention. The term “study” refers to one or more articles reporting on one intervention.

The following items were captured on the extraction form:

- Study characteristics
- Carer sample size
- Carer demographics
- Primary diagnosis of care recipient
- Intervention description
- Follow-up time
- Outcomes reported among carers
- Implementation outcomes
- Reported effectiveness of intervention (excluding qualitative and pre-post studies)

All data were charted by either ER, NH, or AI. Data on study design, sample size, intervention characteristics and outcomes were checked for accuracy by ER for every study. Quality appraisal was not conducted, in line with standard methodology for scoping reviews (Levac et al. 2010).

Intervention characteristics were charted and analyzed following the Template for Intervention Description and Replication (TIDieR) checklist and guide (Hoffmann et al. 2014). Through ongoing discussions and thematic analysis of intervention descriptions in NVivo 14, we categorized interventions into three overarching approaches to aid analysis. Outcomes related to the implementation of the intervention were classified according to Proctor et al.’s taxonomy of implementation outcomes (Proctor et al. 2011).

Reported effects were captured for each reported outcome for completed intervention studies with a comparison group (excluding pre-post studies and qualitative studies). For each outcome, a statistically significant improvement in favor of the intervention (Yes/No) was recorded.

### Consultation with experts by experience

We conducted two patient and public involvement (PPI) workshops with 18 carers of people with breathlessness (11 women and 7 men, aged 35–78) in May 2024. Participants supported people with diagnoses including heart failure, COPD, asthma and long COVID, and had caring responsibilities for between three months and 30 years. Participants were ethnically diverse with 10/18 selecting a non-white ethnicity. In these workshops we invited carers to prioritize different areas of need and reflect on the challenges they face that are most relevant to them. Learnings from these workshops guided our interpretation of the data by identifying the types of support carers would most like to receive and the outcomes that are relevant to them. We did not solicit specific feedback on the review in these sessions. We held a further workshop with three carers of people with COPD in May 2025 in which we presented findings from the review and invited participants to interpret them. This workshop shaped our analysis of results, guided our focus on which findings to expand further in our discussion, and shaped our recommendations for research and practice. A report on the methods and findings from the workshops is available in supplemental material.

## Results

### Search results

**Figure 1.**
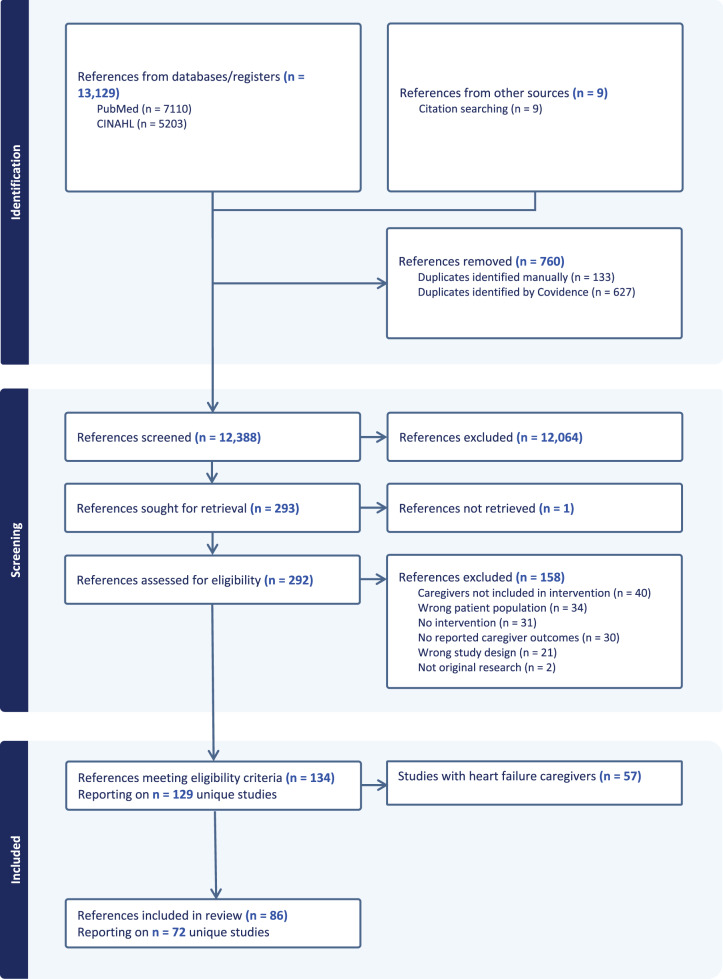
PRISMA Diagram.

Searches returned 12,396 publications after removing duplicates. Nine publications were identified through other search methods. Two hundred and eighty-five publications proceeded to the full-text screening phase, and 129 publications were found to meet inclusion criteria. Eighty-six publications reporting on 72 interventions for carers of people with lung cancer, COPD, ILD or mixed diagnoses presenting with breathlessness are included in this analysis (Fig. 1 – PRISMA Diagram) (Douglas et al. 2005; Kurtz et al. 2005; Lobchuk et al. 2006; Richardson et al. 2007; Cornwall et al. 2008; Hudson et al. 2008; Ryan et al. 2008; DuBenske et al. 2010, 2014; Lindell et al. 2010, 2018, 2021; Porter et al. 2011; Sladek et al. 2011; Cox et al. 2012; Halldórsdóttir and Svavarsdóttir 2012; Namkoong et al. 2012; Smothers and Buck 2012; Bastian et al. 2013; Horton et al. 2013; Zakrisson et al. 2013; Utens et al. 2014; Badr et al. 2015; Borneman et al. 2015; Marques et al. 2015a, 2015b; Milbury et al. 2015a, 2015b, 2019, 2018, 2020; Sun et al. 2015, 2017, 2018, 2019; van den Hurk et al. 2015; Barton 2016; Carson et al. 2016; Figueiredo et al. 2016; Farquhar et al. 2016; Kayyali et al. 2016; Mosher et al. 2016, 2017, 2019; Ellis et al. 2017; Nguyen et al. 2017, 2018; Schellekens et al. 2017; van Manen et al. 2017; Ateş et al. 2018; Campbell and McErlane 2018; Pooler et al. 2018; Stellefson et al. 2018; Winger et al. 2018; Wittenberg et al. 2018; Hasuo et al. 2019; Li et al. 2019; Siouta et al. 2019; Aboumatar et al. 2020; Broese et al. 2020; Frith et al. 2020; Lafaro et al. 2020; Tapia and Campos 2020; Xiu et al. 2020; Dean et al. 2021; Prieto et al. 2021; Schunk et al. 2021; Amin et al. 2022; Ferrell et al. 2022; Gazzi et al. 2022; Grosbois et al. 2022; Kuzu and Aydın 2022; Song et al. 2022; Vagharseyyedin et al. 2022; van Harlingen et al. 2022; Xiao et al. 2022; Zhu et al. 2022; Zomerdijk et al. 2022; Bahadori et al. 2023a, 2023b; Esmaeili et al. 2023; Shao et al. 2023; Byun et al. 2024; Dusel et al. 2024; Latifah et al. 2024; Leng et al. 2024; Ora et al. 2024).

### Study characteristics

Of the 72 interventions included in this analysis, 29 were evaluated by randomized controlled trials (RCTs), 13 were pre-post studies, 13 were evaluated using qualitative methods only, three were cohort studies, and eight used other experimental designs. Sixteen were pilot studies and eight were study protocols for which corresponding published results could not be found. Further details of included studies are available in supplemental materials.

Median follow-up time for RCTs was three months (1–24 months). Across all study designs 11 studies reported follow-up of at least six months, and seven of 12 months or more.

Studies were conducted in 19 countries. Thirty-two of 72 studies were conducted in North America, and 23 in Europe. Only eight studies were conducted in low or middle-income countries (Iran, Turkey, Thailand, and China).

### Characteristics of included carers

In studies with published results (*N* = 64 excluding eight protocols) 5763 carers of people with breathlessness participated in either intervention or comparison groups. Thirty-five interventions targeted carers of people with lung cancer, 17 COPD, and 15 mixed diagnoses or other. Interstitial lung diseases were least well-represented with five interventions (all for IPF carers). No interventions were found for carers of people living with mesothelioma or lung fibrosis following SARS-CoV2 infection.

Fifty-two of 64 studies with published results reported age of participating carers and 56 of 64 reported gender. The median age of participating carers ranged from 40 to 70 years. There were 3 studies with median carer age < 50, conducted in Iran and Thailand. In all but one study more than half of participating carers or family members were female. Carers were most often spouses/partners or adult children, with other relatives including siblings and grandchildren making up a small minority of participants.

The majority (44/64) of studies with published results did not report the race or ethnicity of carers. About half (36/64) reported the socioeconomic status (SES) or education level of participating carers. No studies reported the sexual orientation of participants. Reporting of all demographic variables was higher among RCTs, although 17/29 published RCTs did not report ethnicity of participants. Nearly all (18/20) studies reporting ethnicity were conducted in the United States, one in the UK, and one in Canada.

Among the 20 studies reporting ethnicity of carers, the median percentage of white participants was 82% (range 63–100%). Most studies reporting ethnicity were conducted with lung cancer carers (14/20). Three studies in the mixed diagnoses group reported a median 65% white participants. One study with COPD carers reported a 97% white sample, and two studies with IPF carers reported an average 96.5% white sample.

### Intervention delivery

Table 2 provides a summary of interventions following the TIDieR framework. A description of each intervention following the TIDieR framework is available in supplemental material.

**Table 2.** Summary of interventions following the TIDieR framework

TIDieR Domain	Number of Interventions (*N* = 72)
Intervention Category (What)	
Education	12
Therapeutic Support	31
Interventions for Patient Management	29
Framework or Theory Reported (Why)	
Yes	38
Interventionist (Who)	
Nurse	22
Specialist	6
Mental Health Professional	7
Multidisciplinary Team	20
Hospice Worker	1
Social Worker	3
Complementary Therapist	6
Peer	1
App or Website	5
Mode of Delivery (How)	
In-Person	41
Remote	19
Remote and In-Person	12
Mode of Delivery (How)	
Group-Based	19
Individual Carer or Dyad	49
Individual and Group-Based	4
Setting (Where)	
Home	26
Primary Care Setting	3
Secondary Care Setting	2
Unspecified Healthcare Setting	33
Healthcare Setting and Home	5
Hospice	2
Community Setting	1
Tailored for Individual Recipients	
Yes	41

Interventions were most often conducted by a nurse or multidisciplinary care team. There were an average of four disciplines represented in multidisciplinary teams (2–9). Teams frequently included nurses, specialist physicians (e.g. oncology, palliative care), physical or occupational therapists, and mental health professionals. Three teams included a social worker. Involvement of a chaplain, dietitian and pharmacist were each reported by one study. Thirty-two studies reported providing training for interventionists.

Most interventions involved both the carer and patient, with only 13 delivered to the carer alone. About one quarter were group-based with a further four interventions offering individual and group components. Nineteen interventions were delivered remotely, either via an app or website or via phone or video call.

Twenty-six interventions were delivered in the carer’s home, including those delivered remotely. Most studies did not provide a detailed description of the intervention setting. Only one intervention was delivered in a community setting.

Over half of interventions included at least some elements that were tailored or responsive to the needs of individual participating carers and/or patients.

### Intervention approaches, frameworks, and outcomes

Three distinct approaches to supporting carers emerged from the data:

1. Education
2. Therapeutic support for the career
3. Interventions for patient management

All interventions aimed to improve outcomes among carers, and the three approaches are distinguished by the pathway through which they aimed to achieve this effect.

### Education

While most interventions included an education component, 12 interventions were distinct in focusing only on carer education. Education programs aimed to improve outcomes among carers by providing information about the patient’s disease and treatment, or training to perform caregiving tasks. These programs usually included information on the patient’s condition, expected illness trajectory, and how to monitor and manage their symptoms.

7/12 studies of education interventions reported a theory or framework used to develop the intervention. These were primarily theories relevant to coping (Lazarus and Folkman’s model of coping, Transactional Model of Stress and Coping) and managing chronic illness (chronic care self-management model, cancer family caregiver experience model).

**Figure 2.**
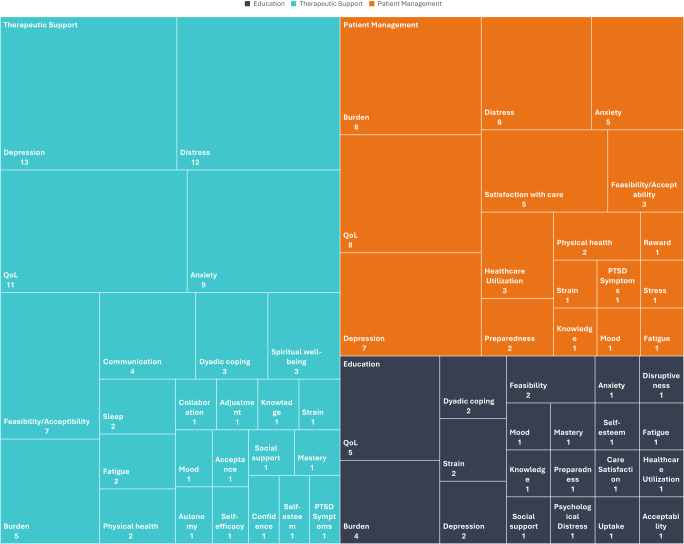
Targeted outcome frequency by intervention category.

The most frequently targeted outcomes for education interventions were quality of life (five studies) and carer burden (four studies). Figure 2 shows the frequency of targeted outcomes across all intervention categories. Education interventions reported significant improvements in outcomes including quality of life, burden, strain, and dyadic coping. One intervention reported an improvement in knowledge of symptom management.

### Therapeutic support

Thirty-one interventions sought to improve outcomes among carers by applying a program of therapeutic support to the carer. Therapeutic support interventions refer to approaches in which a structured therapeutic component, distinct from education, case management, or navigation was delivered directly to the carer or to the carer-patient dyad, with the primary aim of improving carer outcomes. A total of 31 interventions were identified that met this definition. These interventions required active participation from the carer and included modalities such as yoga and other mind-body practices, psychotherapy, coaching, and peer or group-based support.

Fifteen out of 31 studies of therapeutic support interventions reported a framework or theory. There was some overlap with the theories used to develop education interventions with two studies using the chronic care self-management model and one using the transactional model of stress and coping. Other studies used established therapeutic approaches including cognitive behavioral therapy and acceptance and commitment therapy, each reported by one study. There were two Vivekanda yoga interventions and one using mindfulness-based stress reduction. The remainder employed other cognitive theories, while 16 studies reported no guiding theory or framework.

These interventions usually targeted outcomes related to psychological well-being including depression (13 studies), distress (12 studies), and anxiety (9 studies) as well as quality of life (11 studies). Only five studies in this group targeted carer burden, relatively fewer than for other approaches. Studies reported significant improvements in distress, quality of life, burden, depression, anxiety, sleep, dyadic coping, adjustment, collaboration, distress, and self-efficacy.

### Interventions for patient management

Twenty-nine interventions sought to support the carer primarily by changing the way healthcare providers or services engaged with the patient and their carer. Interventions that changed any aspect of how patient care is organized and delivered with carer involvement were classed as patient management interventions. These approaches included palliative care interventions, case management, hospital at home, and carer involvement in breathlessness support services or pulmonary rehabilitation.

These complex interventions offered multiple components, e.g. a comprehensive assessment of the patient’s needs, often with the involvement of the carer, and the development of a personalized care plan. Other common components included those designed to improve coordination between health professionals, such as structured interdisciplinary meetings, and those designed to improve carers’ access to health professionals including a designated point of contact. Many studies in this category looked at the impact of involving carers in psychosocial or disease management programs already available to the patient. Others examined the feasibility or desirability of moving aspects of care into the home through telehealth or hospital at home programs.

Seventeen out of 29 of these studies reported a framework or theory. Five interventions employed a palliative-care based approach, although only 2 referenced a specific model of palliative care delivery used to guide the intervention. Four were based on the program theory for a pulmonary rehabilitation or breathlessness support service. One study used the cancer family caregiver experience model.

These studies targeted diverse outcomes including distress, depression, anxiety, knowledge, and preparedness. The most commonly targeted outcomes were burden (8 studies), quality of life (8 studies), and depression (7 studies). Studies in this category reported significant improvements in quality of life, distress, stress, knowledge, and preparedness.

### Differences across diagnoses

**Figure 3.**
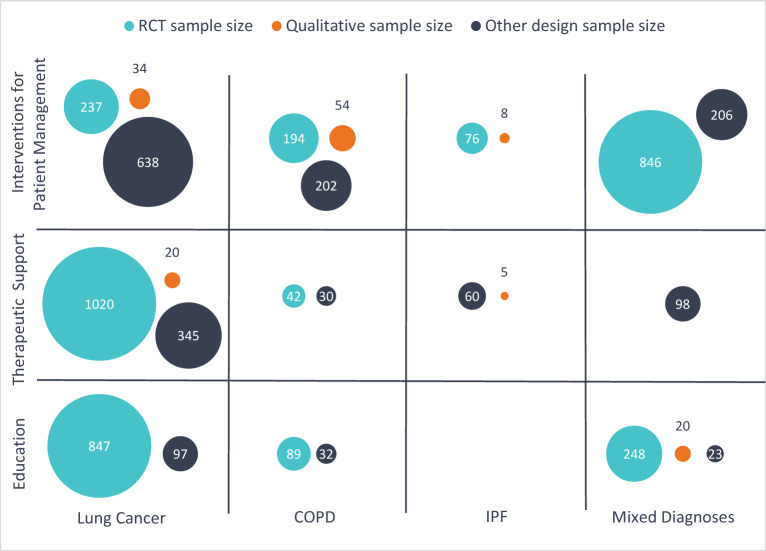
Combined sample size of carers by intervention category, care recipient diagnosis, and study design (excludes study protocols).

Figure 3 shows the combined sample size of carers by study design for each intervention type and diagnostic group. Education and therapeutic support interventions have mainly been studied among lung cancer carers, while interventions for patient management were more evenly distributed between groups. There has been relatively little research testing therapeutic support interventions for COPD carers. There were more studies of interventions for patient management for COPD carers than for other groups, but the combined sample size of lung cancer carers was larger.

## Discussion

Recognizing the essential role of unpaid carers in society, the European Care Strategy for carers and care receivers calls for member states to include in their national action plans for long-term care plans for “supporting informal carers, who are often women and relatives of care receivers, through training, counselling, psychological and financial support” (European Commission 2022). This review synthesized data from 72 studies involving 5,763 carers of patients experiencing breathlessness. There was a dominance of research focusing on carers of people with lung cancer (56% of participants). Studies were found on the effectiveness of strategies to improve many of the outcomes identified in previous research as most important to this population of carers including those relating to mental well-being, supporting symptom management, care team coordination, and social support. We found a surprising lack of studies testing therapeutic support interventions for COPD carers, despite these carers having especially high unmet support needs for their physical and psychological well-being (Blütgen et al. 2025). Notably, most studies lacked comprehensive demographic reporting.

All three intervention approaches identified have the potential to improve outcomes that are important to carers of people with breathlessness. Therapeutic support and patient management interventions frequently targeted outcomes related to mental well-being such as anxiety, depression and distress, which are especially prevalent in this group of carers and were identified as priorities by attendees at our PPI workshops. Some therapeutic support interventions included tools for the carers to support communication with the patient, addressing a key challenge identified by carers we spoke with who identified many challenges in their relationship with the person they support. Many patient management interventions explicitly aim to improve collaboration between the care team and between the care team and the unpaid carer. Carers who attended our PPI workshops felt strongly that better communication with health providers was important to help them carry out their role as carers successfully. Education interventions may meet an expressed need of carers of people with breathlessness for more information on symptom management and what to expect in the future (Caress et al. 2009; Chen et al. 2016; Lee et al. 2020). However, it is important that these interventions are tailored to the information needs of individual carers or groups of carers. Interventions of all types delivered in a group setting may have the added benefit of providing opportunities for connection and peer support, another expressed priority for this group of carers. Future studies should examine the efficacy of each approach at improving the outcomes that matter most to carers, with particular attention to which intervention components are most effective at improving knowledge, mental well-being, and communication between patients, carers and health providers.

We found few interventions for COPD carers relative to the global burden of COPD. These carers have high unmet support needs including the need for mental health support (Badr et al. 2017), information about symptom management, and better communication with health providers. Carers who attended our priority-setting workshops also expressed a desire for more support in these areas and emphasized how the distressing nature of breathlessness leads to stress and constant vigilance. Carers who interpreted the findings of the review suggested stigma as a possible factor in the lack of interventions for COPD carers. They suggested that stigma may cause an inherent bias in research and in the support they are offered compared with other carers, for example those caring for someone with cancer. These carers were not surprised by this finding and reported feeling that COPD is generally dismissed by both professionals and the public.

This review found a lack of evidence on how interventions might address the needs of carers from different ethnic, cultural, or socioeconomic backgrounds due to a lack of demographic reporting and diversity in included studies. Reporting of carer demographics was inconsistent even among RCTs. In some cases, studies reported detailed demographic information for patients but almost none for carers, despite reporting outcomes for carers. Most studies did not report the ethnicity of participating carers, limiting the ability to generalize findings. It is crucial that researchers collect and report this data where it is legal to do so. Many studies that enrolled the patient and carer together also did not clearly identify which intervention procedures or outcome measures were applied to the carer, the patient, or both. People with lower incomes and those from global majority ethnic groups experience higher rates of breathlessness than wealthier or white people, and it is crucial that carers from these communities are represented in research. Currently there is no Equator network standard for the reporting of interventions that enroll patients and carers together, and we strongly recommend the development of such a standard to ensure consistency in reporting. The issue of unclear reporting of dyadic interventions was also highlighted by a recent systematic review (Blütgen et al. 2025).

Most interventions were delivered face-to-face, making them resource and time intensive, which may limit their scalability and cost-effectiveness. Carers who attended our PPI workshops reported that, in the absence of professional support, they often seek out information on symptom management online. Many web-based resources that provide information about breathlessness and the conditions that cause it have not been formally evaluated and were thus beyond the scope of this review. This includes the website “Supporting Breathlessness” (2020) which was co-produced with unpaid carers and people experiencing breathlessness (Farquhar et al. 2017). Evaluations of the reach and effectiveness of these resources are urgently needed. Future research should focus on better understanding the potential of digital interventions for carers, particularly those for whom digital approaches are culturally acceptable and align with their level of digital literacy. There may also be a need to co-design interventions that specifically aim to enhance digital knowledge and literacy among carers of people living with breathlessness. This approach aligns with the UK government’s agenda to digitize health and social care services (NHS England 2025).

### Strengths and limitations

This is the first review to look holistically at interventions to support carers of people with breathlessness, and to compare the volume and type of interventions across conditions that cause breathlessness. This study is strengthened by consultation with carers which impacted our selection of data to extract and interpretation of findings. A limitation of our PPI involvement was that the findings of the review were only interpreted by COPD carers. This review has several limitations. First, it focused solely on interventions designed for carers of people living with breathlessness due to respiratory disease, meaning interventions available to a general population of unpaid carers were not included. Additionally, the exclusion of carers of advanced cancer patients other than lung cancer may have excluded interventions relevant to carers of breathless people. Some interventions classified under mixed diagnoses might have encompassed carers of individuals with COPD, lung cancer, or interstitial lung disease without clear specification, risking an undercount of interventions available for these groups. Our search was limited to studies published after 2000, limiting the number of potentially eligible studies but potentially increasing the relevance of findings for contemporary health services. Finally, as a scoping review no meta-analysis was conducted. Included studies cannot be assumed to be adequately powered to detect changes in outcomes among participating carers, and those studies which did not report significant effects were not necessarily ineffective. Further research is needed to determine which interventions are most effective to improve which carer outcomes. We have identified types of interventions and groups of carers where there is sufficient evidence for a future meta-analysis to be conducted, as well as areas where more primary studies are needed. In particular, we recommend more trials of therapeutic support services for COPD carers, and more research into all types of support for carers of ILD patients.

## Conclusion

This review advances prior work by shifting the focus from documenting carers’ unmet needs to mapping supportive interventions, revealing uneven research attention across diseases and identifying priorities for equitable intervention development. We identified three approaches to supporting carers of people with breathlessness: Education, therapeutic support, and interventions for patient management. There is likely sufficient evidence for future reviews of the effectiveness of each approach at improving carer mental health, quality of life, and self-efficacy. We found relatively few interventions targeting COPD carers and ILD carers given the global burden of these conditions, and no interventions specifically targeting carers of people living with mesothelioma or fibrotic lung disease following SARS-COV2 infection. We found a lack of evidence on how interventions may affect carers from different backgrounds due to lack of demographic reporting and low ethnic diversity in studies which did report this. These gaps in evidence limit our ability to translate intervention strategies to new populations and settings. To improve equity, researchers must consistently report carer demographics and prioritize developing interventions for carers that have been underserved by research.

## Supporting information

10.1017/S147895152510148X.sm001 Rochester et al. supplementary material Rochester et al. supplementary material

## Supplementary material

The supplementary material for this article can be found at https://doi.org/10.1017/S147895152510148X.
