# Supplementary material for: Interventions for unpaid carers of people living with breathlessness due to chronic respiratory diseases: Scoping review
Source: Palliat Support Care. 2026 Feb 13;24:e58. doi: 10.1017/S147895152510148X (PMC13166304; doi:10.1017/S147895152510148X)
Supplement: Rochester et al. supplementary material [file S147895152510148Xsup001.zip › S147895152510148Xsup001/Findings from PPI Workshops with Unpaid Caregivers of People with Breathlessness (1).docx]

# Research Priorities of Unpaid Caregivers of People with Breathlessness: Findings from three Patient and Public Involvement Workshops

## Aim

To identify common unmet needs and experiences of caregivers of people with breathlessness due to varied diagnoses, and to assess their priorities for research and support.

## Workshops

We conducted two priority-setting PPI workshops with carers of people with breathlessness in May 2024. 18 caregivers attended (11 women and 7 men), aged 35-78. Participants looked after people with diagnoses including heart failure, COPD, asthma and long COVID, and had caring responsibilities for between 3 months and 30 years. Participants in these virtual workshops represented all regions of England and were ethnically diverse with 10/18 selecting a non-white ethnicity. Not all identified with the term “caregiver” with some preferring “supporter” or identifying simply as the patient’s spouse.

In addition to open discussion, participants completed a ranked choice voting exercise to prioritize areas of need for future research.

A graphic artist attended both workshops and created an artwork representing the causes participants identified for their feelings of anxiety, distress and overwhelm (Figure 1).

We held a further discussion with three carers of patients with COPD in April 2025. In this workshop we asked participants to reflect on the findings of a scoping review that found many existing interventions to support carers of people with breathlessness, but relatively few for COPD carers.

## Findings

Caregivers identified anxiety and disruption to daily life as common aspects of their experience. Watching a loved one struggle to breathe is a profoundly distressing experience and participants described a feeling of hypervigilance, worrying that each breath “could be their last”. The unpredictability of breathlessness, which can improve or worsen suddenly, disrupted participants’ ability to make both short and long-term plans for the future.

Participants in all workshops described shame and stigma related to smoking or perceived smoking behavior as an important part of their experience supporting someone with breathlessness. Participants felt that this stigma impacted their treatment by health professionals, particularly in the context of COPD. Participants in the later 2025 workshop identified stigma as a potential reason for the lack of interventions to support COPD caregivers. They described minimal engagement from health professionals, who often exclude them from discussions about patient care and do not proactively signpost them to resources. These carers are left on their own to seek out information online.

In a ranked choice voting exercise, participants selected the following items as their top priorities for research and support:

1. Understanding my relative’s illness
2. Greater recognition and respect from professionals

Participants also identified an unlisted third priority of psychological support. They suggested that opportunities to connect with caregivers in similar situations would be a potentially effective approach.

## Next Steps

Actions that would be responsive to the priorities identified include:

1. Identify existing resources to educate caregivers of people with conditions causing breathlessness about the underlying condition and about breathlessness as a symptom and develop strategies to signpost carers who may benefit to these resources.
2. Provide opportunities for caregivers of people with breathlessness to connect with one another, either through removing barriers to existing services or co-designing new in-person or remote support networks.
3. Identify effective strategies to reduce anxiety among carers of people with breathlessness.
