# Supplementary material for: Interventions for unpaid carers of people living with breathlessness due to chronic respiratory diseases: Scoping review
Source: Palliat Support Care. 2026 Feb 13;24:e58. doi: 10.1017/S147895152510148X (PMC13166304; doi:10.1017/S147895152510148X)
Supplement: Rochester et al. supplementary material [file S147895152510148Xsup001.zip › S147895152510148Xsup001/Support for Carers of People with Breathlessness Scoping Review Protocol (2).docx]

# Forms of Support for Family Members and Informal Caregivers of People with Breathlessness: Scoping Review Protocol

Authors: Eleanor Rochester, Glenn Robert, Irene J Higginson

Reviewed and agreed by: Nikel Hector-Jack, Assma Ibrahimi, Charles Reilly

# Objectives

1. To map the available evidence on forms of support available to caregivers of people with breathlessness with respect to the types of interventions studied and their context, the sizes and designs of studies, and the populations of caregivers that have been included and excluded.
2. To identify evidence gaps and unanswered questions about the best ways to support carers of people with breathlessness that warrant further research.

# Review Question:

What evidence is available describing or evaluating forms of support for adult carers and family members of people with breathlessness due to advanced disease?

- 1. What conditions and types of patients (those supported by carers) were included?
  2. In which settings were interventions conducted (ie. Hospital, community, hospice, care home).
  3. What methodologies have been used to study the effectiveness of forms of support for caregivers?
  4. How often do studies report demographic and socioeconomic information about participants?
  5. Which demographic groups of caregivers have been included in the research? Which family relationships have been included in research?
  6. How often are carers of people with breathlessness explicitly identified as a population of interest in research?

# Background

Census data estimates that just under one in 10 people in the UK are Informal Caregivers, almost half of whom provide ≥20 hours of care a week (Carers UK, 2022). Carers in England and Wales contribute £162 billion per year to the economy, the value of unpaid care being equivalent to a second NHS (Petrillo, Bennett, and Pryce, 2022).

In this review we will use the definition of carers from Carer’s UK: A carer is a person of any age who provides unpaid care and support to a family member, friend or neighbor who is disabled, has an illness or long-term condition, or who needs extra help as they grow older. This broad definition includes anyone who looks after a loved one, whether or not they identify as a caregiver.

Despite their key role, informal carers are often not well-supported by health and social care services (Aoun et al., 2021). Carers are often ambivalent to considering their own needs – three quarters do not self-identify as carers, with many family members acting as carers (Carers UK 2022) and some feel the term diminishes the significance of their responsibilities, limiting the reach of interventions (Carduff et al. 2014). While self-help interventions for those who are ill have increased, those for their families and carers have not kept pace. Interdisciplinary research in breathlessness has not yet included informal caregivers. The voices of minoritized caregivers are particularly underrepresented in research. In addition to improving their wellbeing and reducing health inequalities, better supporting carers may help to keep people at home, reducing pressures on hospitals.

Breathlessness is a frightening and common symptom experienced by people with long-term health conditions (Maddocks et al. 2019). It is experienced by over two million people in the UK with chronic lung or heart disease, and many more with cancer and neurological diseases. Breathlessness has a devastating impact on patients and their families, permeating their lives and relationships, especially during crises (Malik, Gysels, and Higginson 2013). Worsening breathlessness intersects with social and support networks, poverty, and healthcare services, to increase the burdens on those who are ill and their carers (Higginson et al. 2020; Lippiett et al. 2019).

Additionally, unplanned Emergency Department (ED) visits by people experiencing a breathlessness-related crisis place significant strain on already depleted healthcare resources in the UK. One in five people presenting to the ED in the UK are there because of breathlessness (UK 2024). Family members and carers play a key role in deciding when a person with breathlessness should present to the ED (Hutchinson, Galvin, and Johnson 2020; Hutchinson et al. 2017), however the factors that contribute to these decisions have not been examined. Research focusing exclusively on patients is not enough; insight into informal caregiver’s knowledge and beliefs about breathlessness is essential to design services that meet the needs of patients and carers alike.

Research that relates to the experiences of informal carers of people with breathlessness has never been synthesized in a review. The aim of this review is to assess the available evidence on (a) the experiences of caregivers of people with breathlessness in their caring role, with a particular focus on their knowledge and beliefs about breathlessness, and (b) the effectiveness of different forms of support that are available to them. The findings of this review will be used to help inform a research priority-setting exercise conducted with carers of people with breathlessness.

# Methods

# **Eligibility Criteria**

**Types of studies:** Any original research design including RCT’s, Quasi-experimental studies, qualitative studies, evaluations

**Types of participants:** Unpaid adult (>18) caregivers or family members of adults with breathlessness or with chronic respiratory illnesses that commonly cause breathlessness. Included conditions are COPD, lung cancer, interstitial lung diseases including pulmonary fibrosis, heart failure, mesothelioma, chronic fibrotic lung disease following COVID-19 infection.

**Types of interventions:** Any intervention that includes caregivers by design including breathlessness support services or related programs, education or training programs for caregivers, peer support programs or any other intervention or service for unpaid caregivers.

Types of outcome measures: Any outcome reported for caregiver participants including feasibility of the intervention, acceptability to caregivers, acceptability to health and social care professionals, physical or mental health of caregivers, quality of life of caregivers, barriers and facilitators to accessing the intervention for caregivers.

**Other inclusion criteria:**

- Published in English
- Published after 2000
- Studies from all countries eligible

**Exclusion Criteria:**

- Studies that focus exclusively on carers for people with conditions that do not commonly cause breathlessness (ie dementia) will be excluded.
- Studies focusing on carers for people with asthma or chronic hyperventilation syndrome will be excluded.
- Studies on support or training for paid home carers or health aides will be excluded.
- Commentary pieces, editorials and opinion pieces
- **Psychometric studies whose focus is the validity of a survey instrument or other measurement tool in a specific population**

# **Search methods for identification of studies**

**Databases:** PubMed, CINAHL, Medline

**Other methods for identification of studies:**

- Screening lists of studies included in evidence reviews listed in NICE Guidelines 150 “Supporting Adult Carers”
- Screening reference lists of included studies to identify further potentially eligible studies
- Open Access theses and dissertations
- Prospero and OSF protocol registrations
- Searching Google Scholar

**Anticipated search period:** Database searches will be conducted in April 2024. Gray literature searches will be conducted in April and possibly May 2024.

**Limiters:** After 2000, Published in English

**Search terms:**

| **Caregivers** | **People with Breathlessness** | **Interventions** |
| --- | --- | --- |
| Caregiver  Informal caregiver  Unpaid caregiver  Family member  Family  Spouse  Partner | Breathlessness  Dyspn?ea  Shortness of breath  Breathing difficulties  Lung conditions  COPD  Chronic Obstructive Pulmonary Disease  Lung Cancer  ILD  Interstitial Lung Disease  Cardiovascular disease  Coronary artery disease  Heart failure  Congestive heart failure  Pulmonary fibrosis  Pulmonary hypertension | Breathlessness support service  Pulmonary rehabilitation  Education  Training  Intervention  Peer support  Support group  Community-based support  Support |

**Screening Methods:**

All records will be screened by two reviewers at both the title/abstract and full-text stages.

Regarding any potential conflicts that arise, we will have frequent conflict meetings to ensure that relevant papers are excluded, and all team members agree.

**Data collection and analysis**

We will create a data extraction form in Microsoft Excel to be used by all reviewers. Data extraction will be conducted by two reviewers for the first 10% of included papers. Conflicts will be discussed and resolved, with adjustments to the data extraction form if necessary. Thereafter data will be extracted by a single reviewer.

**Items to extract from included studies:**

| **Study Characteristics** | **Intervention Characteristics and Outcomes** |
| --- | --- |
| Author/year  Country/region  Aims  Study design  How participants were identified (inclusion criteria)  How participants were recruited  Methods used to collect information from or about carers  Sample size  Follow-up time  Carer demographics   - Age - Gender - Relationship to care recipient - Race/ethnicity. - SES - Sexual orientation and gender identity - Disability and health challenges   Care recipient primary diagnosis | Summary of intervention  Setting of intervention  Interventionist  Theoretical model used to develop intervention  Target population for the intervention: Caregivers, patients, or both?  Outcomes, if relevant and how measured   - Was the intervention feasible (according to the authors)? - Was the intervention acceptable to caregivers? - Did the intervention impact carer’s health, quality of life or some other outcome? - Did the intervention impact the caregiver’s knowledge? - Did the intervention impact the caregiver’s self-efficacy, confidence or ability to adapt to their caring role? |

**Analysis:**

We will map the occurrence of study characteristics (intervention type, setting, methodology) and caregiver/care recipient characteristics (relationship, ethnicity etc.) quantitatively.

An Evidence and Gap Map may be produced depending on the volume and nature of included studies. If it is deemed by the review team that information on study characteristics can be effectively presented through summary statistics, an EGM will not be created.

Quantitative and qualitative findings from included studies that relate to the review aims will be summarized narratively.

Findings that relate to the experiences of caregivers or family members from minoritized groups will be highlighted.

No quantitative synthesis will be conducted as we do not intend to appraise the methodological rigor of included studies.

# Funding

This review is funded by the King’s Together Seed funding scheme and KCL Faculty of Nursing, Midwifery and Palliative Care seed funding scheme. The funders have no role in the development of the protocol or in the conduct of the review.

# Amendments

This protocol was approved by all review authors on 11/4/2024.

Any changes to this protocol after the start of the review will be recorded, along with justification for the change.

**23/4/2024**

Amendment 1 – Clarification on inclusion of systematic and other reviews.

Systematic and other reviews retrieved in the initial search that are identified as potentially containing relevant studies will be saved and their reference lists will be screened for relevant studies. Any potentially relevant citations will be retrieved and proceed to full-text screening.

Systematic reviews that include a meta-analysis that meets all inclusion criteria for this review will be eligible for inclusion. All other reviews will be excluded after their reference lists are screened.

**4/7/2024**

Amendment 2 – Use of the TiDier framework

After beginning data extraction, we have identified the need for a structured approach to collect and analyze information on aspects of the included interventions. The TiDier Checklist has been selected, and we will amend our data extraction form to include all aspects of the checklist and collect this information from included studies where possible.

Aoun, Samar M, Paul A Cafarella, Bruce Rumbold, Geoff Thomas, Anne Hogden, Leanne Jiang, Sonia Gregory, and David W Kissane. 2021. 'Who cares for the bereaved? A national survey of family caregivers of people with motor neurone disease', *Amyotrophic Lateral Sclerosis and Frontotemporal Degeneration*, 22: 12-22.

Carduff, Emma, Anne Finucane, Marilyn Kendall, Alison Jarvis, Nadine Harrison, Jane Greenacre, and Scott A Murray. 2014. 'Understanding the barriers to identifying carers of people with advanced illness in primary care: triangulating three data sources', *BMC family practice*, 15: 1-10.

Carers UK. 2022. "Key facts and figures about caring." In.

Higginson, Irene J., Deokhee Yi, Bridget M. Johnston, Karen Ryan, Regina McQuillan, Lucy Selman, Stephen Z. Pantilat, Barbara A. Daveson, R. Sean Morrison, and Charles Normand. 2020. 'Associations between informal care costs, care quality, carer rewards, burden and subsequent grief: the international, access, rights and empowerment mortality follow-back study of the last 3 months of life (IARE I study)', *BMC Medicine*, 18: 344.

Hutchinson, Ann, Kathleen Galvin, and Miriam J Johnson. 2020. '“So, I try not to go…” acute-on-chronic breathlessness and presentation to the emergency department: in-depth interviews with patients, carers, and clinicians', *Journal of pain and symptom management*, 60: 316-25.

Hutchinson, Ann, Alistair Pickering, Paul Williams, J Martin Bland, and Miriam J Johnson. 2017. 'Breathlessness and presentation to the emergency department: a survey and clinical record review', *BMC pulmonary medicine*, 17: 1-7.

Lippiett, Kate Alice, Alison Richardson, Michelle Myall, Amanda Cummings, and Carl R May. 2019. 'Patients and informal caregivers’ experiences of burden of treatment in lung cancer and chronic obstructive pulmonary disease (COPD): a systematic review and synthesis of qualitative research', *BMJ open*, 9: e020515.

Maddocks, Matthew, Lisa Jane Brighton, Morag Farquhar, Sara Booth, Sophie Miller, Lara Klass, Deokhee Yi, Wei Gao, Sabrina Bajwah, and William D-C Man. 2019. 'Holistic services for people with advanced disease and chronic or refractory breathlessness: a mixed-methods evidence synthesis', *Health Service Delivery Research*, 7.

Malik, Farida A, Marjolein Gysels, and Irene J Higginson. 2013. 'Living with breathlessness: a survey of caregivers of breathless patients with lung cancer or heart failure', *Palliative medicine*, 27: 647-56.

Petrillo, M, M Bennett, and G Pryce. 2022. "Cycles of caring: transitions in and out of unpaid care." In. London: Carers UK.

UK, Asthma and Lung. 2024. "What is Breathlessness?" In.
